# Early worsening of diabetic retinopathy in individuals with type 2 diabetes treated with tirzepatide: a real-world cohort study

**DOI:** 10.1007/s00125-025-06466-8

**Published:** 2025-07-10

**Authors:** Adam J. Buckley, Garry D. Tan, Marta Gruszka-Goh, Peter H. Scanlon, Imran Ansari, Sara G. I. Suliman

**Affiliations:** 1 Imperial College London Diabetes Centre, Abu Dhabi, United Arab Emirates; 2 OCDEM, Oxford University Hospitals NHS Foundation Trust, Oxford, UK; 3 Oxford NIHR Biomedical Research Centre, Oxford, UK; 4 Gloucestershire Hospitals NHS Foundation Trust, Cheltenham, UK

**Keywords:** GIP, GLP-1RA, Retinopathy, Tirzepatide, Type 2 diabetes

## Abstract

**Aims/hypothesis:**

Early worsening of diabetic retinopathy (EWDR) has been described during treatment with glucagon-like peptide-1 receptor agonists including subcutaneous semaglutide. Whether EWDR occurs after initiating treatment with the potent glucagon-like peptide 1 / gastric inhibitory polypeptide receptor agonist tirzepatide is unknown.

**Methods:**

In this retrospective cohort study using real-world clinical data, we matched 3435 tirzepatide-exposed (≥180 days treatment) individuals with type 2 diabetes 1:1 with 3434 tirzepatide-unexposed individuals for sex, diabetes duration, retinopathy status, HbA_1c_, number of retinal screening episodes and use of glucose-lowering medications. New-onset diabetic retinopathy and retinopathy progression were explored using conditional logistic regression.

**Results:**

Individuals included in the study had tight baseline glycaemic control (mean HbA_1c_ 56.1 ± 15.8 mmol/mol [7.28 ± 1.43%]). New-onset proliferative diabetic retinopathy (PDR) (grade R3M0, R3M1) occurred in 1.1% of tirzepatide-exposed (*n*=33) and 0.5% of tirzepatide-unexposed (*n*=17) individuals. Tirzepatide was significantly associated with new-onset PDR in multivariate analysis after adjustment for established risk factors (OR 2.15 [95% CI 1.24, 3.74], *p*<0.01). However, tirzepatide was also associated with reduced odds of new onset of retinopathy (OR 0.73 [95% CI 0.62, 0.86], *p*<0.001) in individuals without diabetic retinopathy (R0M0) at initiation in multivariate analysis, and was not significantly associated with retinopathy progression in individuals with mild non-proliferative diabetic retinopathy (NPDR, grade R1M0 or R1M1).

**Conclusions/interpretation:**

Tirzepatide therapy resulted in significantly increased odds of incident PDR, particularly in individuals with mild NPDR with maculopathy (grade R1M1), or moderate-to-severe NPDR with or without maculopathy (grade R2M0, R2M1). The increase in odds of progression would justify specialist ophthalmologist referral by Early Treatment Diabetic Retinopathy Study (ETDRS) criteria.

**Graphical Abstract:**

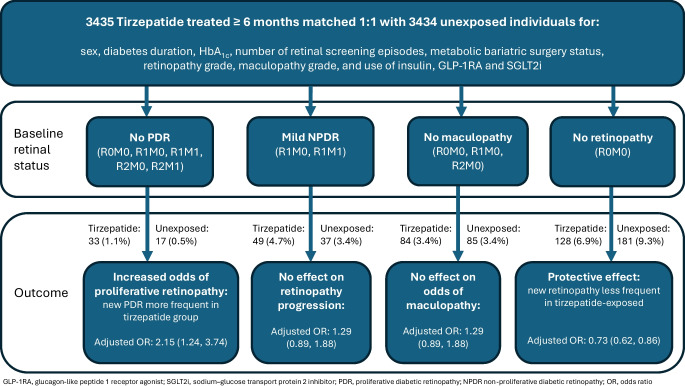

**Supplementary Information:**

The online version of this article (10.1007/s00125-025-06466-8) contains peer-reviewed but unedited supplementary material.


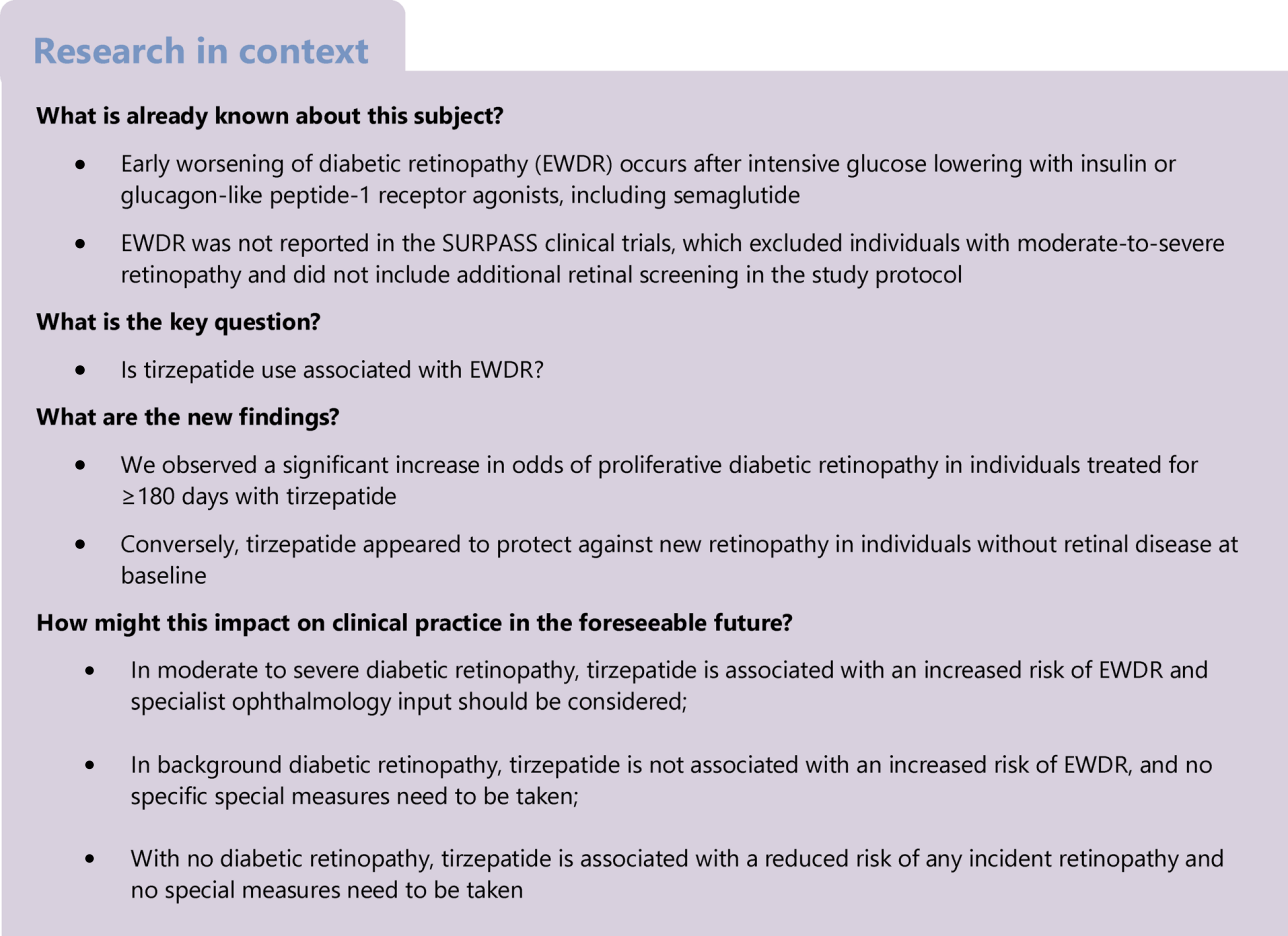


## Introduction

Rapid tightening of glycaemic control in individuals receiving intensive treatment for diabetes can lead to paradoxical early exacerbation of diabetic retinopathy [1]. Early worsening of diabetic retinopathy (EWDR) was first reported in individuals with type 1 diabetes receiving intensive treatment with insulin [2, 3]. While some individuals experience only temporary retinal changes [3], others progress to proliferative diabetic retinopathy (PDR) [4].

EWDR was reported in people with type 1 diabetes undergoing intensive glucose lowering as part of the DCCT trial [5] and has also been described in people with type 2 diabetes in small case–control studies [6]. EWDR also occurs following metabolic bariatric surgery (MBS) [7]. The aetiology of EWDR is poorly understood, although higher baseline HbA_1c_, greater HbA_1c_ reduction, longer diabetes duration and severity of pre-existing retinopathy have been identified as risk factors in meta-analysis [1].

While EWDR was first reported in patients treated with insulin, it is also associated with use of other hypoglycaemic agents, in particular glucagon-like peptide-1 receptor agonists (GLP-1RAs) [8]. Treatment with exenatide was implicated in EWDR in proportion to the reduction in HbA_1c_ [9], although retinopathy status was subsequently stable or improved in the majority of patients who continued treatment [10]. An increased rate of early retinal complications, compared with placebo, was also reported in the SUSTAIN 6 trial of subcutaneous semaglutide in individuals with type 2 diabetes [11].

Tirzepatide is a novel GLP-1RA / gastric inhibitory polypeptide (GIP) co-agonist licensed for treatment of type 2 diabetes [12]. In clinical trials, tirzepatide therapy resulted in HbA_1c_ reductions of as much as 2.58 ± 0.05% (28.2 ± 0.5 mmol/mol), comparable with those seen in intensive glucose-lowering studies. As a consequence, tirzepatide has the potential to increase the risk of EWDR relative to less-effective therapies. Although recent meta-analysis of the SURPASS clinical trials did not detect an increase in risk of EWDR related to tirzepatide treatment [13], these studies did not implement additional retinal screening and their protocols excluded individuals with PDR, severe pre-proliferative retinopathy or maculopathy.

Despite the potential for EWDR, intensive glucose-lowering therapy results in lower rates of retinopathy and better long-term eye outcomes [5, 14, 15]. Although not yet supported by formal guidelines, quarterly eye monitoring has been advocated in individuals at highest risk of EWDR during intensive glucose lowering [1]. Identification of those at risk of EWDR following initiation of tirzepatide will enable appropriate targeting of monitoring and treatment.

## Methods

This retrospective cohort study was performed at Imperial College London Diabetes Centre (ICLDC). ICLDC’s dedicated in-house diabetic retinopathy screening (DRS) service provides two 45° fundus images using mydriatic digital photography annually for all patients with diabetes. All images are examined by primary and secondary licensed retinal graders before final review by a Consultant Ophthalmologist. Patients attending ICLDC have medications resupplied via an in-house pharmacy with all dispensing recorded in the electronic medical record (EMR), allowing close monitoring of medication adherence.

### Data collection

Data were collected from EMRs under the terms of ICLDC’s prospective consent for use of anonymised patient data in research. Inclusion criteria were age ≥18 years, diagnosis of type 2 diabetes, and at least one episode of gradable diabetic retinopathy screening in the 24 months prior to, and one episode after, 24 October 2022, the date when tirzepatide became available for prescription at ICLDC. Exclusion criteria were diabetes of other aetiology, lack of gradable retinal images, previous PDR diagnosis or intravitreal administration of anti-vascular endothelial growth factor in the 60 days prior to tirzepatide initiation. Exposure to tirzepatide was defined as at least two recorded episodes of tirzepatide being dispensed, equivalent to ≥180 days’ treatment, with exclusion of individuals who reported never administering their tirzepatide, as previously reported [16]. The ICLDC EMR does not record any socioeconomic information. EMR access for data collection was concluded on 9 August 2024. The study protocol was approved by the ICLDC Research Ethics Committee (ref.: IREC 100).

Retinal images were graded using the English NHS Diabetic Eye Screening Programme grading form [17], which is compared with the Early Treatment Diabetic Retinopathy Study (ETDRS) grading form and the international classification of diabetic retinopathy for retinopathy and maculopathy in electronic supplementary material [ESM] Tables 1 and 2. Each eye was given a retinopathy grade (denoted by ‘R’) and a maculopathy grade (denoted by ‘M’) according to severity level. Individuals with pre-existing mild non-proliferative diabetic retinopathy (NPDR) (graded R1M0) were classified according to whether background changes were present in one eye (R1a) or in both (R1b) for purposes of matching [18]; individuals with only one assessable eye were considered to have R1b if background retinopathy was present [16].

### Matching

Tirzepatide-exposed individuals were matched with tirzepatide-unexposed individuals at a 1:1 ratio using nearest-neighbour propensity score matching (PSM) without replacement using Mahalanobis distance with matching order set to ‘closest’, based upon clinical characteristics on or prior to 24 October 2022. Individuals were matched according to self-reported sex, diabetes duration in years since first type 2 diabetes-related ICD-10 code (https://icd.who.int/browse10/2019/en), mean HbA_1c_ within the 180 days prior to clinical availability of tirzepatide at ICLDC, number of diabetic retinopathy episodes prior to and after the clinical introduction of tirzepatide at ICLDC, MBS status, use of insulin, GLP-1RA or sodium–glucose cotransporter 2 inhibitor (SGLT2i) within the 180 days prior to clinical availability of tirzepatide, and prior retinopathy and maculopathy grade.

We additionally generated matched datasets using coarsened exact matching (CEM) to assess bias; the results obtained from analysis of these alternative datasets did not differ from those obtained using PSM and are therefore not reported.

After matching, the index date for each paired unexposed individual was defined as the date of first tirzepatide dispensing for the matched exposed individual. Clinical variables were then redefined as the value immediately prior to the index date for each matched group. Missing values (5.5%) were multiply imputed using the *mice* package version 3.17.0 (https://cran.r-project.org). Concomitant medication use was defined as ≥90 days of that medication dispensed within the 6 months prior to the index date.

### Endpoints and specified covariates

The pre-specified PDR endpoint was incidence of new PDR (R3M0, R3M1) in individuals without PDR at baseline. The pre-specified retinopathy progression endpoint was defined as either new moderate-to-severe NPDR (R2M0, R2M1) or new PDR (R3M0, R3M1) in individuals with no retinopathy or mild NPDR at baseline (R0M0, R1M0, R1M1). The maculopathy endpoint was incidence of new M1 in individuals without maculopathy at baseline. The new-onset retinopathy endpoint was defined as any grade of retinopathy occurring in individuals without any retinal disease at baseline. Pre-specified covariates included mean pre-treatment HbA_1c_, mean HbA_1c_ after treatment initiation, largest recorded drop in HbA_1c_ post treatment initiation, diabetes duration, mean arterial pressure (MAP), pre-existing retinal status, pre-existing microalbuminuria or albuminuria, LDL-cholesterol and prior use of oral hypoglycaemic agents (OHAs), GLP-1RAs or insulin. Smoking was not included due to lack of clear evidence regarding the direction of association with retinal outcomes in individuals with type 2 diabetes [19]. Follow-up data collection was censored at 550 days after tirzepatide initiation, due to the very small number of individuals followed up beyond this interval.

### Statistical analysis

Statistical analysis was performed using R version 4.3.1 (R Core Team, Vienna, Austria) with the tidyverse, survival, survminer, MatchIt and mice packages. The influence of variables of interest on study endpoints was assessed using conditional logistic regression using robust SEs with additional evaluation of estimate and SEs using bootstrapping. Pre-specified covariates were removed from models where they were not independently significant and did not substantially alter the information criterion, using a combination of manual selection and automated techniques, or where they were also used as matching criteria. Statistical significance was assessed at the level of *p*<0.01 to adjust for multiple pre-specified endpoints.

## Results

### Matching and baseline characteristics of matched dataset

A total of 3435 tirzepatide-exposed individuals were matched with 3434 tirzepatide-unexposed individuals (Table 1). Post-matching standard mean differences were all <0.05, variance ratios for continuous variables were close to 1, and empirical cumulative distribution functions were effectively minimised (ESM Fig. 1, ESM Table 3). Among treated individuals, the mean ± SD maximum tirzepatide dose achieved was 9.96 ± 3.29 mg/week and the average mean tirzepatide dose during the study period was 6.91 ± 2.18 mg/week.

**Table 1:** Baseline characteristics of matched participants

Total	Control participants (*n*=3434)	Treated participants (*n*=3435)
Female	1973 (57.5)	1966 (57.2)
MBS	256 (7.5)	255 (7.4)
R0M0	1963 (57.2)	1902 (55.4)
R1M0	566 (16.5)	558 (16.2)
R1M1	523 (15.2)	495 (14.4)
R2M0	106 (3.1)	171 (5)
R2M1	276 (8)	309 (9)
Any retinopathy	1471 (42.8)	1533 (44.6)
Diabetes duration, years	10.42 ± 7.23	10.59 ± 7.41
Pre-initiation HbA_1c_, mmol/mol	54.9 ± 15.1	57.4 ± 16.5
Pre-initiation HbA_1c_, %	7.17 ± 1.36	7.40 ± 1.49
Post-initiation HbA_1c_, mmol/mol	54.8 ± 15.0	51.8 ± 15.2
Post-initiation HbA_1c_, %	7.16 ± 1.35	6.89 ± 1.37
MAP, mmHg	90.52 ± 11.00	91.00 ± 9.31
Pre-initiation LDL-cholesterol, mmol/l	2.24 ± 0.94	2.28 ± 0.96
Pre-initiation triglyceride, mmol/l	1.68 ± 1.40	1.73 ± 1.30
Pre-initiation weight, kg	81.98 ± 16.01	91.97 ± 17.61
Pre-initiation BMI, kg/m^2^	31.09 ± 5.46	34.41 ± 6.01
Insulin treatment	1177 (34.3)	1190 (34.6)
Sulfonylurea	1386 (40.4)	1222 (35.6)
SGLT2i	2795 (81.4)	2802 (81.6)
DPP4i	1559 (45.4)	940 (27.4)
GLP-1RA	2841 (82.7)	2900 (84.4)
Fenofibrate	68 (2)	80 (2.3)
Pre-existing microalbuminuria	1157 (33.7)	1289 (37.5)

Data are presented as mean ± SD or *n* (%)

Background retinal disease status of the more severely affected eye is reported

DPP4i, dipeptidyl peptidase-4 inhibitor

### Incidence of new PDR (R3M0, R3M1)

New PDR was detected in 33 of 3068 (1.1%) tirzepatide-exposed individuals and 17 of 3168 (0.5%) tirzepatide-unexposed individuals, a combined incidence rate of 6.9 (95% CI 5.3, 9.0) per 1000 person-years. The majority of incident PDR occurred in individuals with moderate-to-severe NPDR with maculopathy R2M1 (tirzepatide, 16; unexposed, 11) or mild NPDR with maculopathy R1M1 (tirzepatide, 13; unexposed, 5). Few events occurred in individuals with mild NPDR without maculopathy (tirzepatide, 1; unexposed, 0), those with moderate NPDR without maculopathy R2M0 (tirzepatide, 1; unexposed, 0) or those with no previously detected diabetic retinopathy R0M0 (tirzepatide, 2; unexposed, 1). The mean ± SD interval to first recorded PDR was 325 ± 143 days in tirzepatide-exposed individuals and 232 ± 131 days in tirzepatide-unexposed individuals.

Tirzepatide exposure was significantly associated with increased odds of new PDR (R3M0 or R3M1) in multivariate conditional logistic regression (OR 2.15 [95% CI 1.24, 3.74], *p*<0.01) adjusted for post-treatment HbA_1c_ (most recent HbA_1c_ in the 180 days after tirzepatide initiation), LDL-cholesterol and MAP (Table 2).

**Table 2:** Association between tirzepatide exposure and new onset of proliferative retinopathy (R3M0, R3M1)

Effect	β	SE	*z* score	OR (95% CI)	*p* value
Tirzepatide exposure	0.766	0.283	2.708	2.150 (1.236, 3.742)	<0.01
Post-initiation HbA_1c_ (mmol/mol)	0.017	0.017	1.019	1.018 (0.984, 1.052)	0.308
LDL-cholesterol (mmol/l)	−0.036	0.181	−0.201	0.964 (0.677, 1.374)	0.840
MAP (mmHg)	0.015	0.019	0.816	1.016 (0.978, 1.054)	0.415

Multivariate conditional logistic regression adjusted for post-initiation HbA_1c_, LDL-cholesterol and MAP

### Incidence of progression of retinal disease in individuals with mild NPDR at baseline (R1M0, R1M1)

New onset of the composite of moderate-to-severe NPDR (R2M0, R2M1) or PDR (R3M0, R3M1) was reported in 49 of 1053 (4.7%) tirzepatide-exposed individuals and 37 of 1089 (3.4%) tirzepatide-unexposed individuals with mild NPDR at baseline (R1M0, R1M1). The combined estimated incidence rate was 34.9 (95% CI 28.2, 42.6) per 1000 person-years. The mean  ±  SD interval to first recorded progression was 255 ± 135 days in exposed individuals and 230 ± 137 days in unexposed individuals. Tirzepatide exposure was not significantly associated with progression to moderate-to-severe NPDR or PDR in this group (OR 1.29 [95% CI 0.89, 1.88], *p*=0.18) (Table 3).

**Table 3:** Association between tirzepatide exposure and incidence of new moderate-to-severe NPDR (R2M0, R2M1) or PDR (R3M0, R3M1) in individuals with R1M0 or R1M1 at baseline

Effect	β	SE	*z* score	OR (95% CI)	*p* value
Tirzepatide exposure	0.258	0.190	1.354	1.294 (0.891, 1.879)	0.175
Post-initiation HbA_1c_ (mmol/mol)	0.018	0.007	2.435	1.019 (1.004, 1.034)	0.015
LDL-cholesterol (mmol/l)	0.275	0.142	1.938	1.316 (0.997, 1.738)	0.053
MAP (mmHg)	−0.031	0.016	−1.929	0.969 (0.989, 1.000)	0.054

Multivariate conditional logistic regression adjusted for post-initiation HbA_1c_, LDL-cholesterol and MAP

### Incidence of any grade of retinopathy in individuals without diabetes-associated eye disease at baseline

During the follow-up period, 128 of 1857 (6.9%) tirzepatide-exposed individuals with R0M0 at baseline, and 181 of 1946 (9.3%) unexposed individuals with R0M0 at baseline, developed new retinopathy R1M0 or above. The combined estimated incidence rate of any new retinopathy in these participants was 70.2 (95% CI 62.8, 78.3) per 1000 person-years. In this group, tirzepatide exposure was associated with significantly reduced odds of new retinopathy in individuals with R0M0 (OR 0.73 [95% CI 0.62, 0.86], *p*<0.001), after adjustment for post-initiation HbA_1c_, LDL-cholesterol and MAP (Table 4).

**Table 4:** Association between tirzepatide exposure and incidence of new retinopathy of any grade in individuals with no retinopathy (R0M0) at baseline

Effect	β	SE	*z* score	OR (95% CI)	*p* value
Tirzepatide exposure	−0.311	0.082	−3.792	0.733 (0.624, 0.861)	<0.001
Post-initiation HbA_1c_ (mmol/mol)	−0.001	0.005	−1.199	0.994 (0.984, 1.004)	0.230
LDL-cholesterol (mmol/l)	0.037	0.066	0.558	1.038 (0.911, 1.182)	0.577
MAP (mmHg)	0.021	0.006	0.327	1.002 (0.990, 1.015)	0.743

Multivariate conditional logistic regression adjusted for post-initiation HbA_1c_, LDL-cholesterol and MAP

### Incidence of new maculopathy

Incident maculopathy was observed in 84 of 2462 (3.4%) tirzepatide-exposed individuals without maculopathy at baseline, compared with 85 of 2447 (3.4%) tirzepatide-unexposed individuals. Tirzepatide exposure was not associated with new-onset maculopathy in adjusted analysis (OR 1.02 [95% CI 0.82, 1.26], *p*=0.87) (Table 5).

**Table 5:** Association between tirzepatide exposure and incidence of new maculopathy (R1M1, R2M1, R3M1) in individuals without maculopathy at baseline

Effect	β	SE	*z* score	OR (95% CI)	*p* value
Tirzepatide exposure	0.017	0.108	0.159	1.017 (0.823, 1.258)	0.873
Post-initiation HbA_1c_ (mmol/mol)	0.001	0.006	0.214	1.001 (0.989, 1.013)	0.831
LDL-cholesterol (mmol/l)	0.103	0.081	1.280	1.110 (0.946, 1.301)	0.200
MAP (mmHg)	−0.000	0.008	−0.061	1.000 (0.983, 1.016)	0.951

Multivariate conditional logistic regression adjusted for post-initiation HbA_1c_, LDL-cholesterol and MAP

### Other potential risk factors

Several other potential risk-modifying factors were explored but were removed from adjusted analyses due to not being independently significant as covariates and not contributing significantly to the information criterion. These included the change from pre- to post-initiation HbA_1c_, pre-initiation triglyceride level and pre-initiation use of other hypoglycaemic agents and fenofibrate.

## Discussion

Tirzepatide is a potent glucose-lowering agent and, at higher doses, provides greater HbA_1c_ lowering than GLP-1RAs including dulaglutide and semaglutide [20, 21]. Our observation that tirzepatide use was associated with PDR but not with diabetic macular oedema is consistent with recent large post-marketing studies of GLP-1RA use [22]. Our finding of increased incidence of PDR in tirzepatide-exposed individuals contrasts with the low rates of PDR reported in the SURPASS 1–5 clinical trials, which reported a total of 21 adverse retinal events in tirzepatide-exposed individuals and 19 events in tirzepatide-unexposed individuals [13]. Although diabetes duration was not an inclusion criterion in the SURPASS trials, individuals enrolled in SURPASS-1 were previously untreated, those in SURPASS-2 were treated with metformin alone, and those in SURPASS-3 were treated with metformin with or without an SGLT2i, while only SURPASS-5 and SURPASS-6 included participants using insulin at baseline, suggesting that the average diabetes duration at inclusion in these trials may have been relatively short. The average incidence rate of any retinal adverse event in these studies was approximately 5.6 per 1000 person-years or 0.6%, lower than the present study and also lower than previously reported in type 2 diabetes cohorts [23, 24]. Importantly, individuals with moderate NPDR or higher did not meet the inclusion criteria for the SURPASS trials and consequently our study is the first to explore retinal outcomes after tirzepatide exposure in individuals at higher risk of retinopathy progression. The difference in retinopathy incidence between the present study and SURPASS also highlights the value of using regular retinal screening, rather than event reporting, in future trials of potent glucose-lowering agents.

Although it has been reported that the risk of EWDR is increased by faster or more effective lowering of HbA_1c_ in individuals treated with insulin or a sulfonylurea [14, 25], in our study reduction in HbA_1c_ was not independently associated with increased odds of the PDR endpoint. A post hoc analysis of the SUSTAIN 6 study found that semaglutide exposure and the degree of reduction in HbA_1c_ at week 16 were independently associated with increased risk of new diabetic retinopathy, with the majority of events contributing to this finding being mild or moderate NPDR [26]. Exposure to exenatide is associated with increased risk of diabetic retinopathy, with the majority of events occurring in individuals in whom HbA_1c_ improved on treatment [9]. In contrast, in the LEADER trial, liraglutide exposure was not associated with a significant increase in retinopathy hazard [11]. A potentially important distinction between these trials is that the mean reduction in HbA_1c_ in LEADER was −0.40%, compared with −1.9% at the 0.5 mg/week dose, and −2.5% at the 1.0 mg/week dose in SUSTAIN 6. The mean HbA_1c_ at baseline in SUSTAIN 6 was 8.7% [11]. In the present real-world study, the mean ± SD change in HbA_1c_ in the tirzepatide-exposed group was −4.6 ± 11.2 mmol/mol (−0.4 ± 1.0%), the mean ± SD HbA_1c_ at baseline was 55.2 ± 15.5 mmol/mol (7.2 ± 1.4%), and a large proportion of tirzepatide-exposed individuals were previously treated with semaglutide. The apparent disparity in our findings is therefore potentially explained by the significantly better baseline glycaemic control of our participants as well as previous exposure to semaglutide [13]. Direct beneficial effects of GLP-1 agonism on retinal vasculature have also previously been reported in animal models [27], which could potentially offer another mechanism by which risks of EWDR associated with rapid HbA_1c_ reduction might be mitigated.

Our study endpoints were ascertained from annual digital retinal photography images and were not synchronised with the date of tirzepatide initiation. Consequently, the CIs for time to progression were large, making it difficult to infer from our data how soon, or how often, individuals at higher risk of progression to PDR should be followed up after initiation of tirzepatide. It should be noted that in the SUSTAIN-6 trial, new diabetic retinopathy events among individuals with pre-existing retinopathy were more frequent among semaglutide-treated individuals from week 0 of follow-up, suggesting that the increase in hazard of EWDR associated with GLP-1RAs may begin at initiation [26]. In the present study, new PDR events occurred in 17 of 113 (15%) of tirzepatide-treated individuals with R2M0 or R2M1 at baseline, which by the ETDRS referral criteria and the 2024 NICE guidelines would justify referral to specialist ophthalmology follow-up. The NHS Diabetic Eye Screening Programme has also recently published criteria for high-risk features of R2, based on ETDRS criteria, which would guide Eye Service referral [10, 17, 26, 28].

### Potential sources of bias

Study participants had a relatively long mean diabetes duration (<10 years) and the majority of individuals in the treatment group were switched to tirzepatide from a GLP-1RA, potentially both limiting the number of endpoints and reducing the applicability of our findings to GLP-1RA naive patients being initiated on tirzepatide. Although this study is based on real-world data in a representative population of people with type 2 diabetes, enhancing its external validity, the improvements in HbA_1c_ seen after introduction of tirzepatide were modest in comparison with those seen in clinical trials and may not be applicable in a population with less-optimised baseline glycaemic control. The use of 1:1 matching, rather than a higher ratio of tirzepatide-exposed to tirzepatide-unexposed individuals, resulted in a loss of statistical power but conversely would tend to reduce the potential for type 1 error. Due to matching for sex and the use of conditional logistic regression for analysis, it was not possible to evaluate the influence of sex on study outcomes.

Strengths of this study include a large study population, enabling good quality of matching and providing sufficient endpoints for multivariate analysis. Other important strengths include the comprehensive consultant-led in-house retinal screening programme at ICLDC, and a relatively consistent 3 monthly cycle of clinic attendance, which enables frequent clinical and laboratory measures and enhances uptake of screening for complications of diabetes.

### Conclusions

Tirzepatide exposure was not associated with progression of retinopathy in individuals with mild NPDR at baseline (R1M0, R1M1) and appeared to reduce the odds of new retinopathy in individuals without retinopathy (R0M0). However, tirzepatide was significantly and independently associated with new onset of PDR, with the majority of events occurring in individuals with mild NPDR with maculopathy (R1M1) or moderate-to-severe NPDR with maculopathy (R1M1 or R2M1). Our findings, in a group of relatively well-controlled individuals with diabetes, not only reinforce the need for additional retinal photography in those already at high risk of PDR who commence treatment with tirzepatide but also provide reassurance that EWDR is rare in individuals with lower pre-treatment risk.

## Supplementary Information

Below is the link to the electronic supplementary material.

Supplementary file1 (PDF 212 KB)

## Data Availability

Data that underlie the results reported in this article after de‐identification (text, tables and figures) will be available. Data will be available for up to 5 years following publication for sharing with researchers who submit a study question which, in the opinion of the authors, can reasonably be addressed by the data. Enquiries should be directed to abuckley@icldc.ae. An institutional contract and data sharing agreement will be required.

## Abbreviations

EMR: Electronic medical record
ETDRS: Early Treatment Diabetic Retinopathy Study
EWDR: Early worsening of diabetic retinopathy
GIP: Gastric inhibitory polypeptide
GLP-1RA: Glucagon-like peptide-1 receptor agonist
ICLDC: Imperial College London Diabetes Centre
MAP: Mean arterial pressure
MBS: Metabolic bariatric surgery
NPDR: Non-proliferative diabetic retinopathy
PDR: Proliferative diabetic retinopathy
PSM: Propensity score matching
SGLT2i: Sodium–glucose cotransporter 2 inhibitor
