# Supplementary material for: Early worsening of diabetic retinopathy in individuals with type 2 diabetes treated with tirzepatide: a real-world cohort study
Source: Diabetologia. 2025 Jul 10;68(9):2069–76. doi: 10.1007/s00125-025-06466-8 (PMC12361273; doi:10.1007/s00125-025-06466-8)
Supplement: Supplementary file 1 — Supplementary file1 (PDF 212 KB) [file 125_2025_6466_MOESM1_ESM.pdf]

Electronic Supplementary Materials

ESM Table 1. Diabetic Retinopathy Classifications of Progression to PDR

ETDRS final Scale	ETDRS (Final) Grade	Lesions	'International' Clinical Classification	English Screening Programme
No apparent retinopathy	10<br>14, 15	DR absent<br>DR questionable		R0<br>Currently screen Annually
Mild NPDR	20	Micro aneurysms only	Ma's only	R1<br>Screen annually<br>**Background**<br>microaneurysm(s)<br>Retinal haemorrhage(s) ± any exudate
	35<br>a<br>b<br>c<br>d<br>e	≥1 of the following:<br>Venous loops ≥ definite in 1 field<br>SE, IRMA, or VB questionable<br>Retinal haemorrhages present<br>HE ≥ definite in 1 field<br>SE ≥ definite in 1 field	More than just micro aneurysms but less severe than Severe NPDR	
Moderate NPDR	43a<br><br>b	H/Ma moderate in 4-5 fields or severe in 1 field or<br>IRMA definite in 1-3 fields		R2<br>Refer to ophthalmologist<br>**Pre-proliferative**<br>venous beading<br>intraretinal microvascular abnormality (IRMA)<br>multiple deep, round or blot haemorrhages
Moderately severe NPDR	47<br>a<br><br><br><br>b<br>c<br>d	Both level 43 characteristics –<br>H/Ma moderate in 4-5 fields or severe in 1 field and IRMA definite in 1-3 fields<br>**or** any one of the following:<br>IRMA in 4-5 fields<br>HMA severe in 2-3 fields<br>VB definite in 1 field	Severe NPDR<br>Any of the following:<br>a) Extensive intraretinal haem (>20) in 4 quadrants<br>b) Definite venous beading in 2+ quadrants<br>c) Prominent IRMA in 1+ quadrant<br>And no signs of PDR	
Severe NPDR	53<br>a<br>b<br>c<br>d	≥1 of the following:<br>≥ 2 of the 3 level 47 characteristics<br>H/Ma severe in 4-5 fields<br>IRMA ≥ moderate in 1 field<br>VB ≥ definite in 2-3 fields		
Mild PDR	61a<br><br>b	FPD or FPE present with NVD absent or<br>NVE = definite		R3<br>Urgent referral to ophthalmologist<br><br>**Proliferative**<br>new vessels on disc (NVD)<br>new vessels elsewhere (NVE)<br>pre-retinal or vitreous haemorrhage<br>pre-retinal fibrosis ± tractional retinal detachment
Moderate PDR	65a<br><br>b	1) NVE ≥ moderate in 1 field or definite NVD with VH and PRH absent or questionable or<br>2) VH or PRH definite and NVE < moderate in 1 field and NVD absent	Neovascularisation<br>Vitreous / preretinal haemorrhage	
High risk PDR	71<br>a<br>b<br>c<br>d	Any of the following:<br>1) VH or PRH ≥ moderate in 1 field<br>2) NVE ≥ moderate in 1 field and VH or PRH definite in 1 field<br>3) NVD = 2 and VH or PRH definite in 1 field<br>4) NVD ≥ moderate		
High risk PDR	75	NVD ≥ moderate and definite VH or PRH		
Advanced PDR	81	Retina obscured due to VH or PRH		

ESM Table 2. Maculopathy Classification

<table>
<tr><th>Early Treatment Diabetic Retinopathy Study</th><th>International classification</th><th>English Classification</th><th>Grade in the English NHS DESP</th></tr>
<tr><td></td><td></td><td>Absence of features below</td><td>M0</td></tr>
<tr><td></td><td rowspan="2">Diabetic Macular Oedema Present as defined by some retinal thickening or hard exudates in the posterior pole and subclassified into:

Mild Diabetic Macular Oedema:
Some retinal thickening or hard exudates in the posterior pole but distant from the macula</td><td>Circinate or group of exudates within the macula (The macula is defined as that part of the retina which lies within a circle centred on the centre of the fovea whose radius is the distance between the centre of the fovea and the temporal margin of the disc)</td><td>M1</td></tr>
<tr><td></td><td>Any microaneurysm or haemorrhage within 1DD of the centre of the fovea only if associated with a best VA of ≤ 6/12 (if no stereo)</td><td>M1</td></tr>
<tr><td>Clinically significant macular oedema as defined by:</td><td rowspan="2">Moderate diabetic macular oedema:
Retinal thickening or hard exudates approaching the centre of the macula but not involving the centre</td><td>Exudate within 1 disc diameter (DD) of the centre of the fovea</td><td>M1</td></tr>
<tr><td>A zone or zones of retinal thickening one disc area or larger, any part of which is within one disc diameter of the centre of the macula.</td><td rowspan="2">Retinal thickening within 1DD of the centre of the fovea (if stereo available)</td><td rowspan="2">M1</td></tr>
<tr><td>Retinal thickening at or within 500 microns of the centre of the macula</td><td rowspan="2">Severe diabetic macular oedema:
Retinal thickening or hard exudates involving the centre of the macula</td></tr>
<tr><td>Hard exudates at or within 500 microns of the centre of the macula, if associated with thickening of the adjacent retina (not residual hard exudates remaining after disappearance of retinal thickening)</td><td></td><td></td></tr>
</table>

ESM Table 3.

Effect of matching on comparability of baseline characteristics between treated cases and controls. DRS = digital retinal screening, R1a = monocular background retinopathy, R1b = binocular background retinopathy. Call = matchit(Tz_exposed~Sex + Dm_duration + mean_HbA1c + Num_episode_Pre + Num_episode_Post + MBS_status + Pre_BG_Ret_Status + Prior_Maculopathy + Insulin + GLP1 + SGLT2i, method = "nearest", distance = "mahalanobis", m.order = "closest", replace = FALSE, data = Match_source, ratio = 1)

	**Before Matching**				**After Matching**			
	**Means Treated**	**Means Control**	**Std. Mean Diff.**	**eCDF Mean**	**Means Treated**	**Means Control**	**Std. Mean Diff.**	**eCDF Mean**
**Sex = Female**	0.573	0.520	0.106	0.052	0.573	0.574	-0.004	0.002
**Sex = Male**	0.427	0.480	-0.106	0.052	0.427	0.426	0.004	0.002
**Diabetes duration (years)**	10.590	10.233	0.048	0.008	10.590	10.414	0.024	0.004
**HbA1c (%)**	7.415	7.062	0.272	0.035	7.415	7.370	0.034	0.006
**DRS episodes pre**	4.422	4.429	-0.004	0.003	4.422	4.378	0.025	0.005
**DRS episodes post**	1.384	1.330	0.108	0.009	1.384	1.371	0.025	0.002
**MBS status**	0.074	0.042	0.122	0.032	0.074	0.074	0.000	0.000
**R0**	0.669	0.707	-0.082	0.039	0.669	0.669	-0.001	0.000
**R1a**	0.226	0.215	0.025	0.010	0.226	0.225	0.001	0.000
**R1b**	0.085	0.064	0.076	0.021	0.085	0.085	0.000	0.000
**R2**	0.002	0.003	-0.020	0.001	0.002	0.002	0.000	0.000
**R3**	0.018	0.010	0.060	0.008	0.018	0.018	0.000	0.000
**M1**	0.236	0.184	0.123	0.052	0.236	0.235	0.002	0.001
**Insulin use**	0.347	0.215	0.278	0.132	0.347	0.344	0.007	0.003
**GLP-1RA use**	0.844	0.336	1.404	0.509	0.844	0.828	0.046	0.017
**SGLT2i use**	0.816	0.688	0.329	0.127	0.816	0.814	0.004	0.001

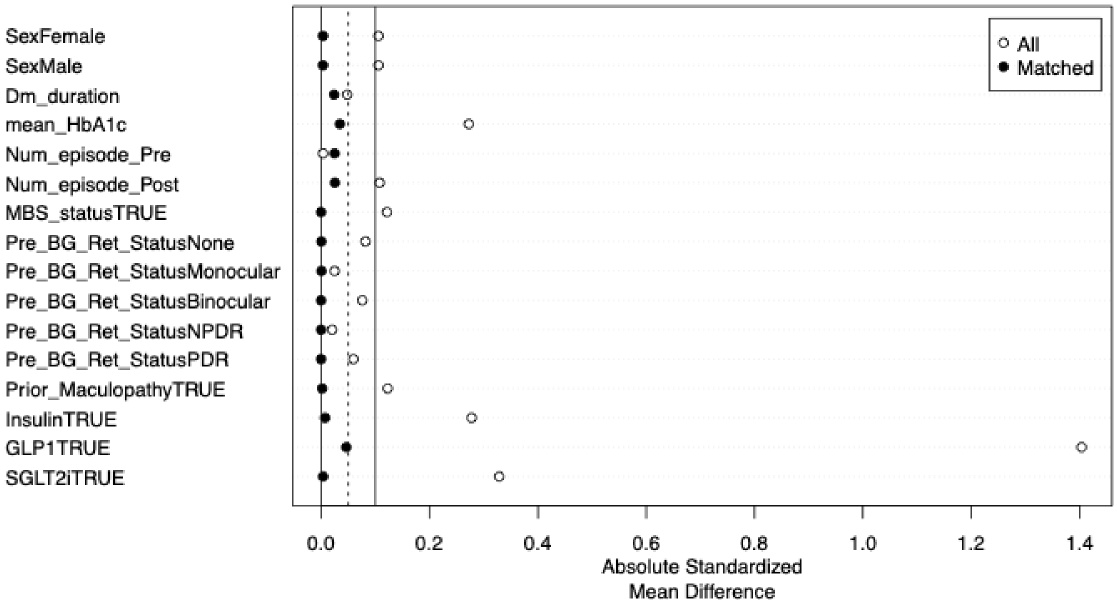

ESM Figure 1: improvement in standardised mean difference following nearest-neighbour propensity score matching Call = matchit(Tz_exposed~Sex + Dm_duration + mean_HbA1c + Num_episode_Pre + Num_episode_Post + MBS_status + Pre_BG_Ret_Status + Prior_Maculopathy + Insulin + GLP1 + SGLT2i, method = "nearest", distance = "mahalanobis", m.order = "closest", replace = FALSE, data = Match_source, ratio = 1)
